# A KLF4/PiHL/EZH2/HMGA2 regulatory axis and its function in promoting oxaliplatin-resistance of colorectal cancer

**DOI:** 10.1038/s41419-021-03753-1

**Published:** 2021-05-13

**Authors:** Xuan Deng, Fanyang Kong, Si Li, Haoqin Jiang, Liu Dong, Xiao Xu, Xinju Zhang, Hong Yuan, Ying Xu, Yimin Chu, Haixia Peng, Ming Guan

**Affiliations:** 1 Department of Laboratory Medicine, Huashan Hospital, Shanghai Medical College, Fudan University, Shanghai, 200040 China; 2 Department of Gastroenterology, Changhai Hospital, Second Military Medical University, Shanghai, 222300 China; 3 Department of Clinical Laboratory, The First Affiliated Hospital of Dalian Medical University, Dalian, 116011 China; 4 Digestive Endoscopy Center, Tongren Hospital, Shanghai Jiaotong University School of Medicine, Shanghai, 200050 China

**Keywords:** Colorectal cancer, Long non-coding RNAs

## Abstract

Long noncoding RNAs (lncRNAs) have emerged as a new class of regulatory molecules implicated in therapeutic resistance, yet the mechanisms underlying lncRNA-mediated oxaliplatin resistance in colorectal cancer (CRC) are poorly understood. In this study, lncRNA P53 inHibiting LncRNA (PiHL) was shown to be highly induced in oxaliplatin-resistant CRC cells and tumor tissues. In vitro and in vivo models clarified PiHL’s role in conferring resistance to oxaliplatin-induced apoptosis. PiHL antagonized chemosensitivity through binding with EZH2, repressing location of EZH2 to *HMGA2* promoter, and downregulating methylation of histone H3 lysine 27 (H3K27me3) level in *HMGA2* promoter, thus activating HMGA2 expression. Furthermore, HMGA2 upregulation induced by PiHL promotes PI3K/Akt phosphorylation, which resulted in increased oxaliplatin resistance. We also found that transcription factor KLF4 was downregulated in oxaliplatin-resistant cells, and KLF4 negatively regulated PiHL expression by binding to PiHL promoter. In vivo models further demonstrated that treatment of oxaliplatin-resistant CRC with locked nucleic acids targeting PiHL restored oxaliplatin response. Collectively, this study established lncRNA PiHL as a chemoresistance promoter in CRC, and targeting PiHL/EZH2/HMGA2/PI3K/Akt signaling axis represents a novel choice in the investigation of drug resistance.

## Introduction

Poor response to chemotherapy is one of the top challenges patients are facing in advanced cancer care. However, the molecular mechanism underlying this complex event has not been fully elucidated. Platinum-based antineoplastic drugs are among the widely used first-line agents for treating solid tumors^1^. By forming drug-DNA adducts, platinum is able to produce DNA lesions that impair DNA replication and transcription, which would eventually cause cell cycle arrest and apoptosis^2^. The third-generation platinum analog, oxaliplatin, is with promising activity in treating colon and rectal cancer^3^. The combination of oxaliplatin plus 5-fluorouracil (5-FU)/leucovorin (FOLFOX) has improved response rates to more than 50% and median survival time approaching 2 years for patients with metastatic colorectal cancer (CRC)^4^. Unfortunately, 40–50% of stage II and stage III CRC patients have shown chemo-resistance to oxaliplatin-based adjuvant therapy and have recurrent disease over the course of treatment^5^. Thus, there is an unmet need for a deep understanding of the molecular mechanisms that contribute to chemotherapy resistance.

Long non-coding RNAs (lncRNAs) are transcripts from non-protein-coding regions of the genome with more than 200 nucleotides (nt) in length^6^. They have been shown to modulate transcription, translation, and post-transcriptional modification of mRNAs and regulate the cellular process involved in cancer pathogenesis^7,8^. P53 inHibiting LncRNA (PiHL) was a newly identified lncRNA located on human chromosome 8. Our previous study revealed that PiHL had three exons with a full length of 599 nt^9^. We clarified that PiHL upregulation is an independent predictor for poor prognosis of CRC patients in two independent cohorts, and PiHL was able to promote proliferation and inhibit apoptosis of CRC in vitro and in vivo^9^. It is generally acknowledged that “futile” attempts to repair DNA damage generated by cytotoxic drugs usually finish in cell death activation. Therefore, changes in key regulators of cell death (which might be a major culprit for drug resistance) often compromise the efficacy of chemotherapy^10^. Indeed, gene expression affected by PiHL silencing was significantly enriched in the cell apoptotic process in our mRNA profile of CRC cells (GSE124526), prompting us to further explore the role and mechanism of PiHL in drug resistance.

In this study, we demonstrated a novel KLF4/lncRNA-PiHL/EZH2/HMGA2 signaling axis that mediated the response of CRC cells to oxaliplatin treatment. We furthermore provided strategies to reverse such resistance in CRC.

## Materials and methods

### Patient sample

In this study, fresh CRC tissues were collected from 100 CRC patients by surgical resection (Shanghai Jiao-tong University School of Medicine affiliated Tongren Hospital). All patients were selected based on a clear pathological and imaging diagnosis with stage II and stage III CRC. None of the patients had received any prior anticancer treatment but underwent oxaliplatin-based adjuvant chemotherapy after surgery. Treatment response was assessed with morphologic imaging (CT) and classified according to the response evaluation criteria in solid tumors guideline into complete response, partial response, stable disease, and progressive disease. Written informed consent for using tumor tissues and clinical data in this study was obtained from each patient. This research was approved by the Ethics Committee of Shanghai Jiao-tong University.

### Cell lines and cell culture

Human CRC Caco-2, RKO, and DLD-1 cells were purchased from the Chinese Academy of Sciences Cell Bank or ATCC. Human CRC SW480, HCT116, and HT-29 were gifts from Dr.Fanyang Kong’s lab. All cells were authenticated by STR profiling (Genetic Testing Biotechnology, Suzhou, China). Patient-derived cancer cells C4 and R21 were isolated from discarded CRC specimens, with patients’ written informed consent and approval of the ethical committee of Tongji Hospital of Shanghai Jiao-tong University School. Briefly, tumor resections were manually minced and digested for 1 h with 0.1% trypsin (Invitrogen, Carlsbad, CA, USA) and 10 U/ml of DNase I (Promega, Madison, WA, USA) in a 37 °C Innova 40 temperature incubator (New Brunswick Scientific, Edison, NJ, USA), followed by a red blood cell lysis using ACK lysis buffer (Beyotime, Shanghai, China). Tissues then were washed and triturated bypassed through a 100 μm cell strainer. Oxa-resistant CRC C4R, R21R, 480R, and 116R cells were induced from C4, R21, SW480, and HCT116 cells, respectively. Cells were cultured in DMEM medium (Gibco, Grand Island, NY, USA), supplemented with 10% fetal bovine serum (Gibco) at 37 °C in an atmosphere with 5% CO_2_. Culture environments were tested and certified as mycoplasma-free.

### RNA isolation and quantitative RT-PCR analyses

RNA isolation was performed as previously described^9^. Briefly, total RNA from the CRC tissue specimens and cell lines was extracted using TRIzol reagent (Invitrogen, Carlsbad, CA, USA), and reverse transcribed using PrimeScript™ RT reagent Kit with gDNA Eraser (Takara, Beijing, China). SYBR® Premix Ex Taq™ GC (Takara) was used for qPCR with primers listed in Supplementary Table 1. Expression levels were calculated relative to β-actin and normalized to control samples.

### Western blot

Western blot analysis was performed as previously described^9^. Cells were suspended in RIPA lysis buffer (Beyotime, Shanghai, China) containing a protease inhibitor cocktail (Sigma-Aldrich, St. Louis, MO, USA). Cell lysates or retrieved proteins were analyzed by immunoblot with primary antibodies and HRP-conjugated secondary antibodies.

### Plasmid construction and cell transfection

Full-length PiHL cDNA was cloned into pCDH-CMV-puro (short for pCDH) vector (System Biosciences, Palo Alto, CA, USA) as previously described^9^. PiHL promoter was amplified from 1000 bp to 1 bp upstream of PiHL’s TSS. These constructs of truncated PiHL promoter were subsequently cloned into the pGL3 vector.

Cells seeded on the plate overnight were transfected with plasmids as indicated in figure legends using Lipofectamine® 3000 (Thermo Fisher Scientific, Waltham, MA, USA) transfection reagent following the manufacturer’s protocol. Cells were harvested at 48–72 h post-transfection for future experiments.

### RNA interference

SiRNA oligonucleotides targeting PiHL, HMGA2, and KLF4 are listed in Supplementary Table 2 (Biotend, Shanghai, China). Cells were transfected with the indicated siRNAs using Lipofectamine 3000 Reagent (Invitrogen, Carlsbad, CA, USA) or Lipofectamine RNAiMAX Reagent (Invitrogen), according to the manufacturer’s protocol. After transfection for 48 h, the cells were used for RNA extraction, CCK8, flow cytometry, apoptosis, and immunoblotting assays. ShRNAs for PiHL were purchased from Biotend.

### Subcellular fractionation

Separation of nuclear and cytosolic fractions was performed using the PARIS Kit (Ambion, USA) according to the manufacturer’s instructions. Cytoplasmic and nuclear fractions were split for RNA and protein extraction.

### CCK-8 assay

CRC cell viability was evaluated with the Cell Counting Kit 8 (MedChemExpress, NJ, USA) and was measured at OD 450 nm with an automatic microplate reader (BioTek, Winooski, VT, USA).

### Flow cytometry analysis

Flow cytometry analysis was performed as mentioned before^9^. Briefly, For the cell apoptosis assay, A total of 1 × 10^6^ cells were resuspended in a single cell suspension and washed two times with PBS solution. The cell apoptosis analysis was performed with the Annexin V-FITC or Annexin V-APC Apoptosis Detection Kit (BD Biosciences, San Diego, CA, USA) according to the manufacturer’s instructions. The rates of apoptosis were detected by flow cytometry (CyAn, Beckman Coulter).

### Colony formation assay

Cells were seeded (1 × 10^3^ cells per well) in a six-well plate and cultured for 14 days. The resulting colonies were then washed twice with PBS and fixed with 100% methanol for 15 min and stained for 30 min with 0.1% crystal violet. The number of colonies was then captured by an Olympus camera (Tokyo, Japan) and counted by ImageJ (NIH, Bethesda, MD, USA).

### EdU incorporation assay

Each group of isolated tumor cells was seeded onto 96-well plates in triplicate at a density of 10^3^/well and incubated for 48 h. Subsequently, the cells were incubated for an additional 2 h in the respective media containing 50 μM EdU (RiboBio, Guangzhou, China). Cell proliferation was detected using a Cell-LightTM EdU Cell Proliferation Detection Kit (RiboBio, China) following the manufacturer’s instructions. DNA was incubated with Hoechst 33342 stain (100 μl/well) for 30 min and visualized using an inverted fluorescence microscope (Nikon 80i Eclipse, Japan). For each EdU experiment, five random fields were imaged at 100× magnification. Captured images were processed and analyzed using ImageJ software. The number of EdU-positive cells was determined by Hoechst nuclear staining and expressed as a percentage of the total number of cells in each field.

### Establishment of oxaliplatin-resistant cells

Oxaliplatin-resistant CRC cells were established as described before^11^. Briefly, oxaliplatin IC50 values of HCT116, SW480, C4, and R21 cells were tested using CCK8 assay. Cells were cultured in a medium with a gradually increased concentration of oxaliplatin (starting with 1/50 IC50). Each dose was maintained for two weeks. Stable drug-resistant cell lines 116R, 480R, C4R, and R21R were selected and cultured with final drug concentration for at least 6 months.

### Fluorescence in situ hybridization (FISH) and immunohistochemistry (IHC) analysis

For combined FISH and IHC, FISH was first performed using Ribo^TM^ Fluorescent In Situ Hybridization Kit (RiboBio) according to the manufacturer’s specifications. Briefly, cells were plated onto coverslips until 60–70% confluent, washed with PBS, and fixed in 4% paraformaldehyde permeabilized with 0.3% TritonX-100. Cells were incubated with PiHL-specific FISH Probe Mix (RiboBio) and washed with saline-sodium citrate (SCC) buffer. Cells were then incubated with primary anti-HMGA2 antibodies followed by incubation with FITC-conjugated goat antibodies against rabbit IgG (Abcam, Cambridge, UK). Cell nuclei were counterstained with 40,60-diamidino-2-phenylindole (DAPI, Invitrogen). Slides were then imaged via fluorescence microscopy (Nikon 80i Eclipse, Japan).

IHC analysis was conducted to determine KLF4 (1:500, ab215036; Abcam), Cleaved-Caspase-3 (1:400, #9661; CST, New England Biolabs, Ipswich, MA, USA), HMGA2 (1:400, #8179; CST) and phospho-Akt (Ser473) (1:100, #4060; CST) protein expression in CRC as described before^12^. Briefly, tissues from xenograft models were embedded in paraffin, cut into 5-mm sections. After deparaffinization and rehydration, 3% hydrogen peroxide is then used to block endogenous peroxidase activity. Tissue slides were next incubated with the indicated primary antibodies overnight. Envision Dual Link System-HRP DAB kit (Dako, Carpinteria, CA) was used to detect antibody binding. Ten fields within the tumor area were randomly selected under 400× magnification for assessment and scoring of immunohistochemical data.

### RNA pulldown assay

RNA pulldown assay was performed as previously described^9^. Full-length or antisense LncRNA PiHL was transcribed in vitro and biotinylated using RNAmax-T7 kit (RiboBio, Guangzhou, China). RNAs were then refolded using Annealing Buffer for RNA Oligos(Beyotime). Three mg RNA pulldown ready PiHL or antisense was incubated with 1 mg nuclear protein extracts from indicated CRC cells, and incubated with Dynabeads Myone Streptavidin T1 beads (Invitrogen). Proteins enriched by beads were then eluted and detected by immunoblots.

### RNA immunoprecipitation (RIP)

The RIP experiment in this study was carried out using the EZ-Magna RIP RNA-binding protein immunoprecipitation kit (Millipore, Billerica, MA, USA) following the manufacturer’s manual. Anti-EZH2 antibodies were purchased from Millipore (5 mg; 07-689). RNAs enriched in each group were detected by quantitative real-time PCR (qRT-PCR).

### Promoter analysis

Potential KLF4-binding sites on 1-kb region directly upstream of PiHL TSS were analyzed via online tool JASPAR (http://jaspar.genereg.net/) and TF prediction program Consite (http://consite.genereg.net/). The Dual-luciferase Reporter Assay kit (Promega) was used to perform a dual-luciferase assay. Briefly, 5000 indicated cells were seeded into a 96-well plate and grow until each well reach 85% confluent. In total, 45 ng constructed pGL3 plasmids (contain various PiHL promoter regions) or pGL3-basic plasmids, combined with 5 ng Renilla luciferase reporter pRL-TK plasmids (internal control), were transfected into each well by Lipofectamine 3000 reagent (Invitrogen). To examine the relationship between KLF4 and PiHL promoter activity, PiHL promoter-reporter plasmid was co-transfected with KLF4 or vector. After 48 h, cells were washed and lysed, Firefly and Renilla luciferase intensity were evaluated by a BD Monolight 3010 luminometer (BD Biosciences, San Diego, CA, USA).

### Mutagenesis assay

Q5 Site-Directed Mutagenesis kit (NEB, New England Biolabs, Beverly, USA) was used to carry out the mutagenesis of EZH2 binding site on PiHL transcript, following the manufacturer’s instruction. Primers for cloning PiHL mutations were designed by NEBase Changer (NEB, http://nebasechanger.neb.com/) and listed in Supplementary Table 1.

### Chromatin immunoprecipitation (ChIP)

ChIP assays were performed using EZ-ChIP kit (Millipore, Boston, MA, USA) according to the manufacturer’s specifications. Chromatin from indicated cells was sonicated to shear DNA and incubated with either with anti-H3K27me3 (ab6002; Abcam), anti-EZH2 (07-689; Millipore), anti-KLF4 (ab215036; Abcam), anti-Histone H3 (#4620, Cell Signaling Technology), normal rabbit IgG (12-370, Millipore) or normal mouse IgG (12-371, Millipore) antibodies. Enrichment of interesting genes or DNA regions was detected by qRT-PCR using specific primers listed in Supplementary Table 1. Fold enrichment was calculated relative to the IgG controls.

### In vivo models

BALB/c-nu/nu athymic nude mice (male, 4–6 weeks) were obtained from Changhai Hospital of the Second Military Medical University and kept in specific pathogen-free (SPF) conditions. No blinding test was used in assessing the outcome. W Oxaliplatin resistance models: 2 × 10^6^ SW480 or 480 R cells were suspended in 0.2 ml PBS and inoculated subcutaneously into the right flank of each mice for tumor formation. Bodyweight and tumor volume was measured every 5 days and recorded in mm^3^ (length × width^2^). When the tumor reached a size of around 200 mm^3^, mice received oxaliplatin or control treatment (intraperitoneal injections, i.p.) every 3 days (*n* = 8). The dosage of oxaliplatin was 5 mg/kg daily.

LNA treatment models: 480 R oxaliplatin-resistant xenografts were established as above mentioned. Two weeks after injection, mice were randomly divided into four groups: Scr_LNA+DMSO, PiHL_LNA+DMSO, Scr_LNA+Oxa, PiHL_LNA+Oxa. In vivo-grade LNA targeting PiHL or scrambled LNA was designed and produced by RiboBio and LNA target sequences were listed in Supplementary Table 2. Mice xenografted were locally injected with in vivo-grade LNAs (50 nmol, twice a week), or intraperitoneal administration of oxaliplatin (5 mg/kg/day), or combination treatment for 4 weeks. In vivo experiments in this study were performed in accordance with relevant guidelines and were approved by Institutional Animal Care and Use Committee at Second Military Medical University.

### Statistical analysis

Statistical analyses were performed with GraphPad_Prism_7.0 (GraphPad Software Inc., CA, USA) or SPSS v.13.0 (IBM, NY, USA) software. Data are presented as mean ± SD of at least three independent experiments. For most of the in vitro and in vivo models, Student’s *t*-test was used to analyze the significance of mean values. IC50 was determined by nonlinear regression “dose-response”. The expression correlation of two genes was determined by Pearson correlation analysis. Kaplan–Meier method and log-rank (one-tail) test were carried out to plot and compare survival curves of mice among different groups. *P*-values < 0.05 were considered statistically significant.

## Results

### PiHL is highly expressed in oxaliplatin-resistant CRC cells and patient tissues

**Fig. 1:**
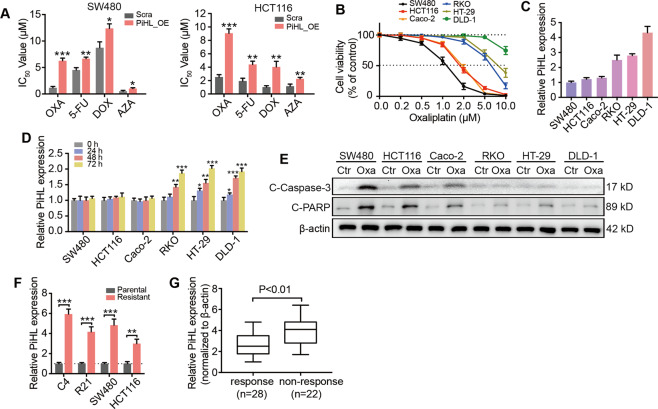
PiHL is activated in oxaliplatin-resistant CRC. **A** IC50 values of OXA, 5-FU, DOX, and AZA in SW480 (left) and HCT116 cells (right) under PiHL overexpression (PiHL_OE) or control (Scra) conditions were determined using the CCK-8 assay. **B** Different CRC cells were treated with increasing concentrations of oxaliplatin for 72 h and exhibit different drug sensitivity. **C** PiHL levels in different CRC cells analyzed by qRT-PCR. **D** Expressions of PiHL in CRC cells treated with 5 μmol/l (μM) oxaliplatin for the indicated time. **E** Immunoblots analyze the expression of cleaved Caspase-3 (C-Caspase-3) and cleaved PARP (C-PARP) after 5 μM oxaliplatin or control (Ctr) treatment for 72 h. **F** Expression of PiHL in Oxa-resistant CRC cells and parental cells was analyzed by qRT-PCR. **G** The expression of PiHL in CRC patients with a response (*n* = 28) or no response (*n* = 22) to oxaliplatin-based therapy from our cohort, two-tailed Student’s *t*-test. Data are presented as the mean ± SD, *P < 0.05, **P < 0.01, ***P < 0.001.

To examine our hypothesis that PiHL upregulation induces cytotoxic drug resistance in CRC cells, we treated SW480 and HCT116 cells with chemotherapeutic cytotoxic drugs now commonly used in clinical settings under either vector or PiHL overexpression conditions. Compared with the control group, PiHL overexpression impaired the cytotoxic effects of oxaliplatin (OXA), 5-fluorouracil (5-FU), doxorubicin (DOX), and azacitidine (AZA) in both SW480 and HCT116 cells (Fig. 1A). Among all the evaluated treatments, PiHL activation showed the most remarkable effects in inducing drug-resistant phenotypes when exposed to OXA, prompting us to further investigate PiHL’s role in mediating OXA resistance.

Seven commercialized colon and rectal cell lines were used to further confirm PiHL’s function in mediating chemo-resistance of oxaliplatin. Cell survival was all reduced when exposed to dose-escalation of oxaliplatin (Fig. 1B). The basal levels of PiHL in RKO, HT-29, and DLD-1 cells were higher than in SW620, SW480, and HCT116 cells, and a significant induced of PiHL expression was observed in RKO, HT-29, and DLD-1 cells following oxaliplatin treatment (Fig. 1C, D). According to immunoblotting of apoptotic associated proteins cleaved-Caspase 3 and cleaved-PARP, HT-29 and DLD-1 cells exhibited lower sensitivity to oxaliplatin-induced apoptosis than SW620, SW480, and HCT116 cells (Fig. 1E).

Next, SW480, HCT116 cells, CRC patient-derived C4 (colon cancer), and R21 (rectal cancer) cells were exposed to cycles of gradually increased concentration of oxaliplatin for at least 6 months to acquire oxaliplatin-resistant phenotypes and were named 480R, 116R, C4R, and R21R, respectively (Fig. S1A). Compared with parental cells, Oxa-resistant cells were less sensitive to oxaliplatin, as shown by increased IC 50 (half maximal inhibitory concentration) value and cell proliferation ability, and reduced cell apoptosis when exposed to oxaliplatin (Fig. S1B–D). The qRT-PCR was performed to compare PiHL expression profiles in different cells and showed that PiHL was significantly activated in oxaliplatin-resistant CRC cells (Fig. 1F).

In our previous study, we showed that PiHL was elevated in CRC tissues compared with normal mucosa in our cohort and TCGA dataset. Further, PiHL levels were examined in 50 stages II and III CRC tissues from patients who received oxaliplatin-based therapy after radical colectomy (Fig. 1G). These results suggested that highly expressed lncRNA PiHL might be involved in the oxaliplatin resistance of CRC cells.

### PiHL is negatively regulated by transcription factor KLF4 in oxaliplatin-resistant CRC cells

**Fig. 2:**
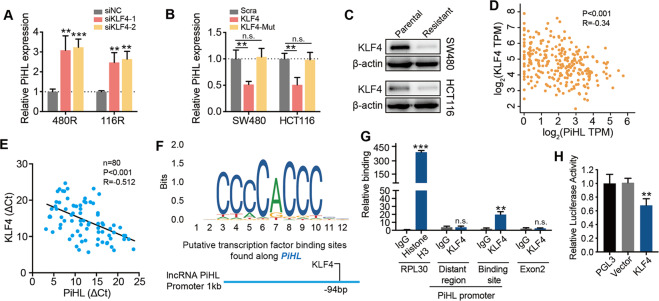
PiHL is transcriptionally regulated by KLF4. **A** Levels of PiHL in KLF4 silencing Oxa-resistant cells were analyzed by qRT-PCR. **B** Levels of PiHL in KLF4 overexpression and DNA binding domain truncated KLF4 mutant (KLF4-Mut) overexpression CRC cells were analyzed by qRT-PCR. **C** Immunoblots showed KLF4 protein levels in parental and oxaliplatin-resistant CRC cells. **D** Correlation between KLF4 and PiHL was generated from GEPIA. *n* = 275 CRC tissues in COAD, Pearson correlation. **E** Correlation between KLF4 and PiHL from our CRC cohort. *n* = 80, Pearson correlation. **F** The transcriptional factor KLF4-binding motif was predicted by informatics analysis (top), schematic illustration of *PiHL* promoter with one potential KLF4-binding site (bottom). **G** ChIP-qPCR assay demonstrated the direct binding of KLF4 to *PiHL* promoter in SW480 cells. **H** Luciferase activity of *PiHL* promoter constructs after the transfection of the KLF4 plasmid in 293T cells. Student’s *t*-test, Significant results were presented as n.s. (non-significant), *P < 0.05, **P < 0.01, ***P < 0.001.

We next explore the potential mechanism for PiHL upregulation in oxaliplatin-resistant CRC cells. No genomic amplification of PiHL gene was detected in the oxaliplatin-resistant cells (Fig. S2A). Inhibition of DNA methyltransferase exerted no influence on PiHL expression in CRC cells (Fig. S2B). To explore whether PiHL expression in Oxa-resistant cells is regulated by its own promoter, possible transcription factors of PiHL were predicted using PROMO and JASPAR online software. RNAi was then used to knock down the candidate transcription factors in SW480R, HCT116R (acquired oxaliplatin resistance), and HT-29 (intrinsic oxaliplatin resistance) cells. It showed that silencing KLF4 (Krüppel-like factor 4) could induce PiHL expression in all three cells (Figs. S2C, D and 2A). Wild type KLF4 transfection inhibited PiHL levels in CRC cells, while DNA binding domain truncated KLF4 mutant (KLF4-Mut) showed no effect on PiHL expression (Fig. 2B). It was reported that colon cancer cells with decreased KLF4 expression are refractory to chemotherapy^13^. In our study, we found that KLF4 expression was significantly inhibited in Oxa-resistant cells compared with parental cells, suggesting that KLF4 might act as a transcription repressor of PiHL in Oxa-resistant CRC cells (Fig. 2C). We next analyzed the correlation of KLF4 levels with PiHL. PiHL was negatively correlated with KLF4 (*P* < 0.001, *R* = −0.34) in TCGA-COAD (Fig. 2D). Consistently, KLF4 mRNA expression was also negatively correlated with PiHL levels in CRC tissues from our cohort (*P* < 0.001, *R* = −0.512; Fig. 2E).

To investigate whether PiHL is a direct target of KLF4, PiHL putative promoter region was predicted using the online software, and one probable binding motif for KLF4 lies −94bp upstream of PiHL transcription start site (TSS) (Fig. 2F). To next determine whether there is direct regulation, we performed ChIP-PCR in Oxa-resistant CRC cells and observed binding of endogenous KLF4 protein to the PiHL promoter KLF4 binding site (−54 to −274) but not to upstream (−751 to −953) nor exon 2 (+316 to +464) (Fig. 2G). In addition, luciferase promoter assays showed that KLF4 reduced PiHL-luciferase activity (Fig. 2H). Thus, PiHL activation in KLF4 knockdown Oxa-resistant CRC cells and interaction between KLF4 and PiHL promoter indicated that KLF4 transcriptionally repressed PiHL expression.

### PiHL antagonizes oxaliplatin-induced apoptosis in CRC cells

**Fig. 3:**
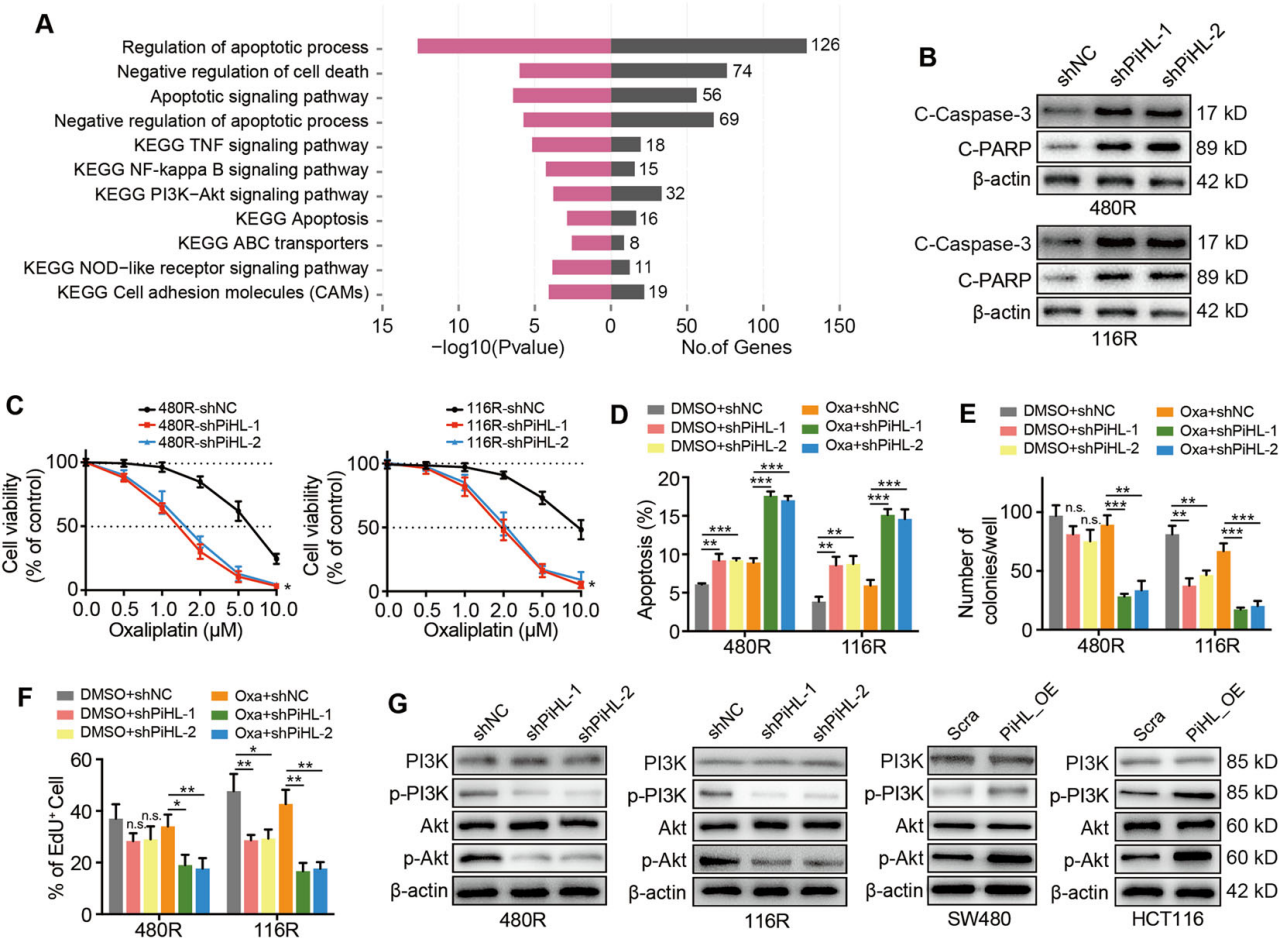
Knockdown of PiHL restores oxaliplatin sensitivity in Oxa-resistant CRC cells in vitro. **A** –Log10 transformations of the *P*-values of top 11 significantly enriched pathways by PiHL knockdown. **B** Expression of cleaved Caspase-3 (C-Caspase-3) and cleaved PARP (C-PARP) in PiHL silencing Oxa-resistant CRC cells after 5 μM oxaliplatin treatment for 72 h were analyzed by western blotting. **C** Effect of PiHL knockdown on Oxa-resistant cells with oxaliplatin treatment at the indicated concentrations for 72 h was analyzed by CCK-8 assay. **D** Flow cytometric analysis revealing the effect of PiHL knockdown on apoptosis of Oxa-resistant cells with oxaliplatin treatment (5 μM) for 72 h. **E** Colony formation of Oxa-resistant cells with PiHL knockdown under oxaliplatin treatment (5 μM) for 72 h. The average number of colonies is shown. **F** EdU assay showing the effect of PiHL knockdown on DNA replication activity of Oxa-resistant cells with oxaliplatin treatment (5 μM) for 72 h. **G** Western blot analysis of indicated proteins in Oxa-resistant and parental CRC cells with PiHL knockdown, PiHL overexpression, or controls. Data are presented as the mean ± SD. *P-*value was determined by Student’s *t*-test or one-way ANOVA. Significant results were presented as n.s. non-significant or *P < 0.05, **P < 0.01, ***P < 0.001.

To further elucidate the functional role of PiHL in oxaliplatin resistance, RNA sequencing was performed to compare transcriptome profiles between PiHL silencing and control HCT116 cells (GSE124526). We observed that PiHL expression was correlated with cell apoptosis, ABC transporters, and PI3K-Akt pathways through GO and KEGG pathway analysis (Fig. 3A). Although ABC transporters were reported to play potential roles in drug resistance^14^, PiHL regulated genes that belong to ABC transporters did not affect cell response to oxaliplatin (Fig. S3A). We then stably knocked down PiHL in Oxa-resistant cells or overexpressed PiHL in parental cells using shRNA or CRIPSR-SAM system, respectively (Fig. S3B, C). The western blotting assay demonstrated that, under oxaliplatin treatment, the levels of apoptosis markers cleaved caspase-3 and cleaved PARP were dramatically increased after PiHL knockdown (Figs. 3B and S3D), while decreased in PiHL-overexpressed SW480 and HCT116 cells (Fig. S3E). Under oxaliplatin treatment, knockdown of PiHL in resistant CRC cells reduced cell viability, induced cell apoptosis, and inhibited colony formation and cell proliferation (Figs. 3C–F and S3F, G). By contrast, PiHL upregulation in parental cells resulted in increased IC50 value, inhibited cell apoptosis, and promoted colony formation and proliferation after oxaliplatin treatment (Fig. S3H, I). In addition, silencing of PiHL inhibited the activation of the PI3K-Akt pathway in Oxa-resistant cells, whereas overexpression of PiHL in SW480 and HCT116 cells promoted PI3K-Akt pathway initiation (Figs. 3G and S3J). Evidence has demonstrated that aberrant activation of the Akt signaling pathway is a frequent event in cancers as well as chemoresistance^15^. Based on the results mentioned above, PiHL might function as part of the PI3K-Akt signaling pathway, which is involved in the oxaliplatin resistance of CRC cells.

### PiHL mediates chemoresistance by promoting HMGA2 expressions

**Fig. 4:**
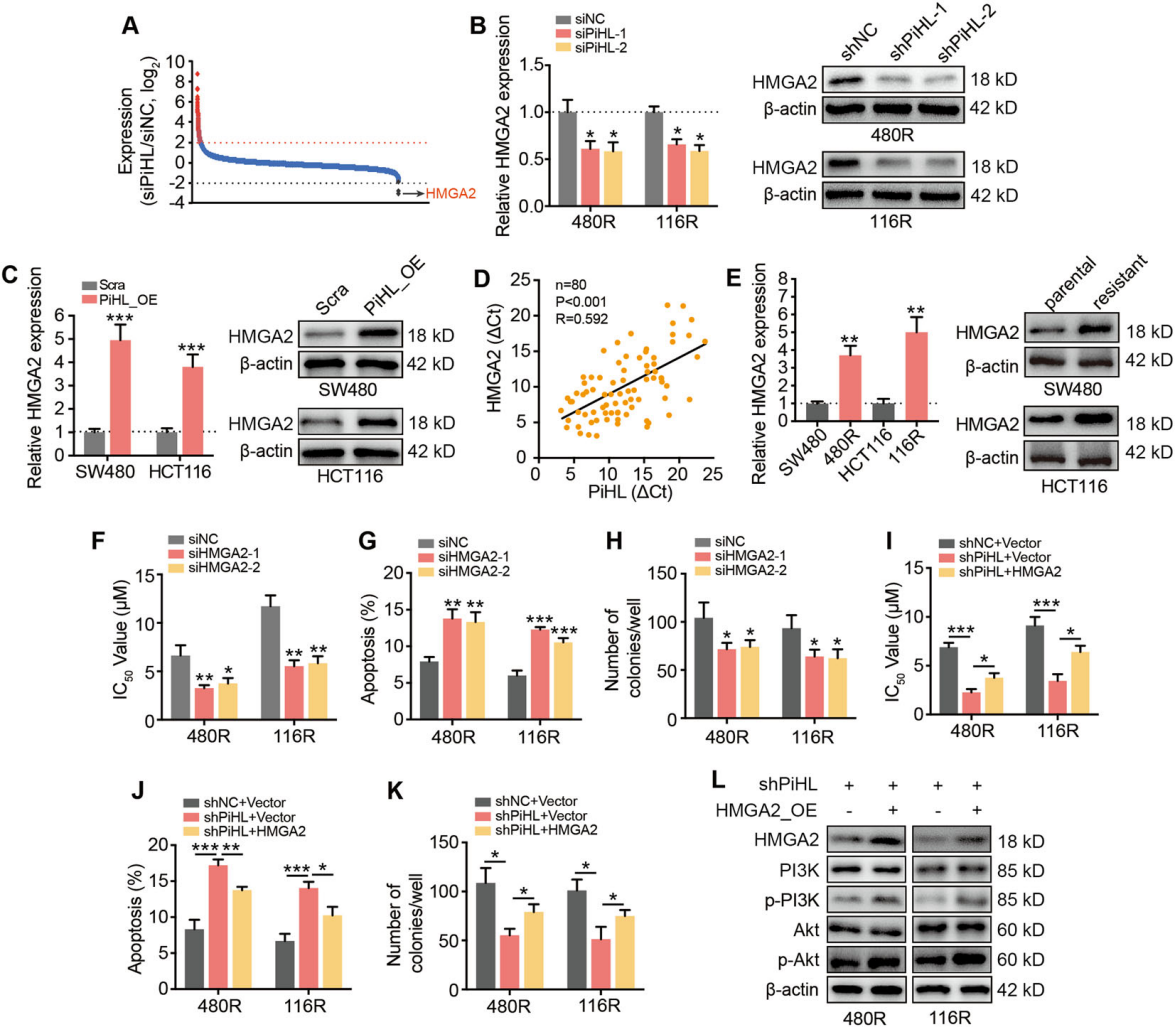
PiHL targeting HMGA2 is required for PiHL-mediated CRC drug resistance. **A** Changes in the expression of cancer-related genes in response to siPiHL treatment compared to siControl are shown. The colored circles indicate genes that are up-regulated (red) or down-regulated (gray) under PiHL-depleted conditions. Changes in the expression of the highlighted genes were experimentally confirmed by qRT-PCR. **B** HMGA2 levels regulated by PiHL in Oxa-resistant CRC cells were confirmed by qRT-PCR (left) and western blotting (right). **C** HMGA2 levels regulated by PiHL in parental CRC cells were confirmed by qRT-PCR (left) and western blotting (right). **D** Correlation between HMGA2 and PiHL from our CRC cohort. *n* = 80, Pearson correlation. **E** HMGA2 expression in Oxa-resistant and parental CRC cells was analyzed by qRT-PCR (left) and western blotting (right). **F**–**H** CCK-8 (**F**), flow cytometry (**G**), and colony formation assay (**H**) were used to determine cell survival, apoptosis, and growth of Oxa-resistant CRC cells with HMGA2 knockdown treated with 5 μM oxaliplatin for 72 h. **I**–**K** CCK-8 (**I**), flow cytometry (**J**), and colony formation assay (**K**) were used to determine cell survival, apoptosis, and growth of Oxa-resistant CRC cells under shNC+Vector, shPiHL+Vector, or shPiHL+HMGA2 conditions treated with 5 μM oxaliplatin for 72 h. **L** Levels of HMGA2 and PI3K/Akt signaling were tested by western blotting in Oxa-resistant cells with indicated treatment. Data are presented as the mean ± SD. *P < 0.05, **P < 0.01, ***P < 0.001.

To further explore the molecular mechanism about how lncRNA PiHL contributes to the chemoresistance phenotype of CRC cells, we selected possible targets of PiHL by analyzing RNA-seq data of PiHL-silencing and control CRC cells (GSE124526). Two hundred and twenty-five genes were dysregulated greater than 2.0-fold in siPiHL-treated cells compared to siControl cells (RPKM > 1, *P* < 0.05) (Fig. 4A). The most down-regulated gene, HMGA2 (High Mobility Group AT-Hook 2), was reported to be involved in the regulation of PI3K-Akt pathway^16^, suggesting PiHL might activate PI3K-Akt via HMGA2. The expression change of HMGA2 by PiHL silencing was confirmed by qRT-PCR and western blotting in oxaliplatin-resistant CRC cells (Figs. 4B and S4A). Conversely, HMGA2 was obviously increased in PiHL overexpressed parental CRC cells at mRNA and protein levels (Fig. 4C). Further, HMGA2 mRNA expression was positively correlated with PiHL levels in CRC tissues from our cohort (*P* < 0.001, *R* = 0.592; Fig. 4D).

HMGA2, which encodes a member of the high mobility group family, was previously shown to be amplified and overexpressed in CRC and contributes to CRC progression^17,18^. Besides, HMGA2 upregulation is known to attenuate cell death under genotoxic drug treatment^19^. As HMGA2 is the target gene of PiHL in CRC cells, we sought to investigate whether HMGA2 mediates PiHL-induced CRC chemoresistance. Significantly higher levels of the HMGA2 mRNA and protein were observed in chemoresistant CRC cells than in the corresponding chemosensitive cells (Fig. 4E). Using gain- and loss-of-function methods, we found that HMGA2 knockdown in chemoresistant cells decreased IC50 values, increased cell apoptosis, and inhibited cell growth (Figs. 4F–H and S4B, C). In contrast, HMGA2 overexpression in chemosensitive cells led to increased IC50 values, reduced apoptosis, and increased cell proliferation (Fig. S4D) after exposure to cytotoxic drugs, indicating that HMGA2 promotes CRC chemoresistance. Further, restoration of HMGA2 recapitulated the oxaliplatin-resistant phenotype and downstream signaling in PiHL-knockdown-resistant cells (Figs. 4I–L and S4E, F). Taken together, the data indicate that HMGA2 signaling is responsible for PiHL-mediated oxaliplatin resistance.

### PiHL epigenetically activates HMGA2 transcription by relieving EZH2 on HMGA2

We then asked how PiHL regulates HMGA2 expression in CRC cells. RNA pull-down assays were first performed and we observed no direct binding between PiHL and HMGA2 (Fig. S5A). In our previous study, we used subcellular fractionation assays to show that PiHL mainly localized in the nucleus^9^. Nucleus-localized lncRNAs are often reported to be epigenetic regulators^20^. Through analysis of the histone modification profile in the UCSC genome browser, we found a high H3K27me3 level in the promoter region of HMGA2, suggesting that PiHL might mediate H3K27me3 on HMGA2 promoter. HMGA2 was previously reported as a regulation target of polycomb group (PcG)^21,22^. It is generally accepted that Enhancer of zeste 2 (EZH2), a subunit of polycomb repressive complex 2 (PRC2), regulates histone methylation by generating histone H3 lysine 27 (H3K27me3) modification on target genes. Recent studies also identify EZH2 as a nuclear lncRNA binding protein. We, therefore, sought to determine whether EZH2 was also a binding partner of PiHL. Interestingly, the online RNA-protein interaction prediction (RPISeq) (http://pridb.gdcb.iastate.edu/RPISeq/) analysis showed a highly possible interaction between lncRNA PiHL and EZH2 protein (random forest classifier value 0.85 and support vector machine classifier value 0.93). Based on this evidence, we hypothesized that PiHL might regulate HMGA2 transcription by interacting with EZH2 protein.

**Fig. 5:**
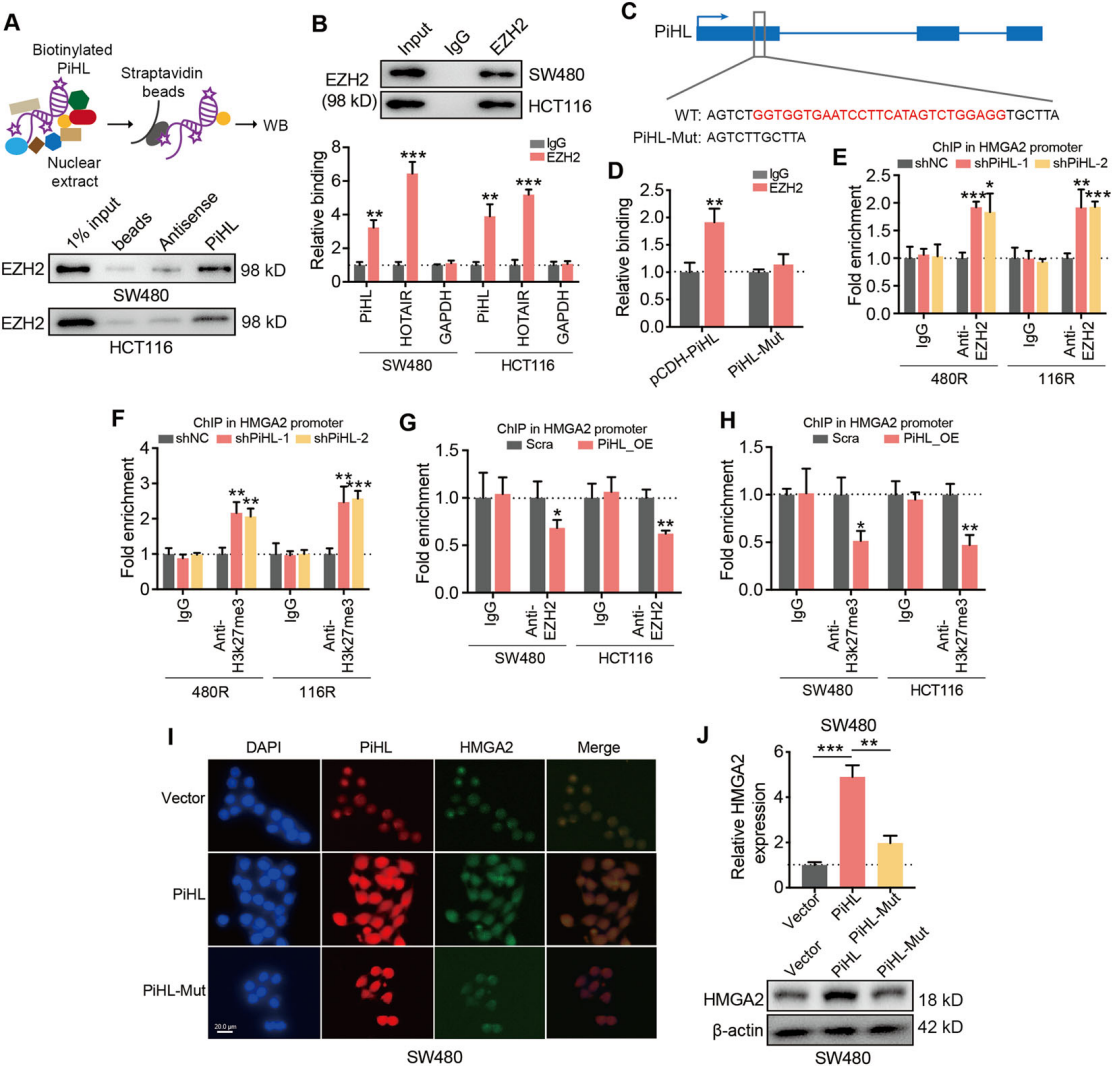
PiHL regulates HMGA2 via interaction with EZH2. **A** Experimental design for pull-down assays (top) and immunoblots of EZH2 proteins retrieved by in vitro-transcribed biotinylated PiHL from cell nuclear extracts (bottom). Antisense PiHL and beads were used as negative controls. **B** Cell nuclear lysates were immunoprecipitated with anti-EZH2 antibodies, Aliquots of cell lysates (10% of input) and IgG or EZH2 immunoprecipitates were separated by SDS-PAGE, and the specific immunoprecipitation of EZH2 was confirmed by Western blot (WB) (top). The complexes were analyzed for the presence of PiHL, HOTAIR (EZH2-binding positive control), or GAPDH (negative control) by qRT-PCR (bottom). Signals were normalized to actin mRNA. **C** Representation of the WT and mutated (PiHL-Mut) PiHL sequences used for immunoprecipitations (IP) with anti-EZH2. PiHL-Mut contains a deletion of the G-quadruple sequence, Fig. S4A. **D** The bar graph shows the relative amount of PiHL after anti-EZH2 IP using lysates of cells transfected with either pCDH-PiHL or PiHL-Mut. **E**, **F** ChIP assay using EZH2 (**E**) and H3K27me3 (**F**) specific antibodies was undertaken in PiHL stably knockdown Oxa-resistant CRC cells to detect the effects of PiHL on EZH2 location and H3K27me3 level at *HMGA2* promoter. **G**, **H** ChIP assay using EZH2 (**G**) and H3K27me3 (**H**) specific antibodies were undertaken in PiHL stably overexpressed CRC cells to detect the effects of PiHL on EZH2 location and H3K27me3 level at *HMGA2* promoter. **I** RNA FISH to visualize PiHL (red) and FITC staining of HMGA2 (green) in SW480 cells transfected with pCDH (Vector, Upper), pCDH-PiHL (PiHL, Middle), or PiHL-Mut (Lower), (Scale bar, 20 μm). Nuclei were stained with 4′,6- diamidino-2-phenylindole (DAPI) (blue). **J** HMGA2 RNA (top) and protein (bottom) levels were measured in SW480 cells transfected with pCDH (Vector), pCDH-PiHL (PiHL), or PiHL-Mut by qRT-PCR and immunoblot, respectively. Data represent the mean ± SD from three independent experiments. *P < 0.05, **P < 0.01, ***P < 0.001.

We then conducted biotin-labeled RNA pulldown followed by western blotting analyses to examine the molecular relationship between PiHL and EZH2. As shown in Fig. 5A, PiHL has interacted with EZH2 in CRC cells. Further, EZH2 is a nucleus-located protein, consistent with the subcellular distribution of PiHL^23^. Subsequently, RIP assays were performed and we found that PiHL was markedly enriched by antibodies against EZH2 (Fig. 5B). For EZH2 binding, lncRNA HOTAIR and GAPDH mRNA were used as positive and negative controls, respectively (Fig. 5B)^24^. PRC2 complex is known to be recruited by G-rich motifs. PiHL sequence was then searched for PRC2-EZH2 potential binding regions and we identified 2 G-quadruple structure motifs (Fig. S5B). Enrichment of a single region (region 1) with 4 GGW repeats by the anti-EZH2 antibody was validated using EZH2-immunoprecipitation (EZH2-IP) combined with qRT-PCR (Fig. S5C). To further investigate whether this GGW region is essential for PiHL to interact with EZH2, a plasmid vector harboring GGW region deletion PiHL mutation (PiHL-Mut) was constructed (Fig. 5C). RIP experiments confirmed that EZH2 failed to interact with PiHL-Mut in HCT116 cells (Fig. 5D).

Next, we explored whether PiHL and EZH2 interaction regulates EZH2 binding and H3K27me3 level at the promoter of HMGA2. ChIP assays showed that silencing of PiHL increased the binding of EZH2 to HMGA2 promoter and induced H3K27me3 level at HMGA2 promoter (Figs. 5E, F and S5D, E). Conversely, ectopic expression of PiHL repressed the binding of EZH2 to HMGA2 promoter and reduced H3K27me3 level at HMGA2 promoter (Figs. 5G, H and S5F). RNA fluorescence FISH of PiHL combined with fluorescein isothiocyanate (FITC) staining of HMGA2 further confirmed PiHL’s nucleus sublocation and suggested that the G-quadruple region is crucial for PiHL to activate HMGA2 expression (Fig. 5I). Consistently, HMGA2 levels reduced in cells transfected with PiHL-Mut compared with wild-type PiHL overexpression cells (Figs. 5J and S5G). Therefore, this evidence indicated that PiHL upregulates HMGA2 expression by relieving the repressive modulation of EZH2 on HMGA2.i

### Knockdown of PiHL restores oxaliplatin sensitivity in vivo

**Fig. 6:**
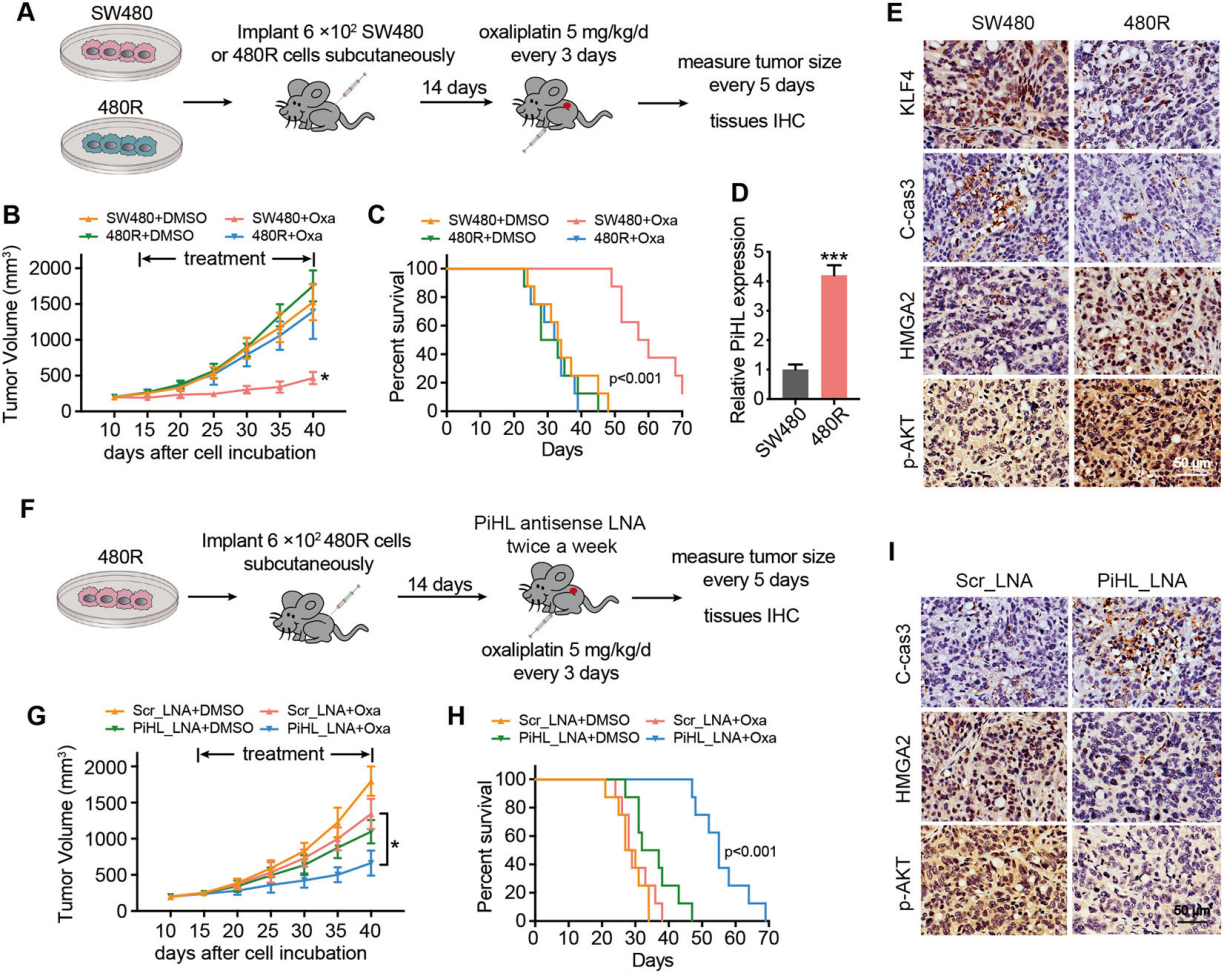
Knockdown of PiHL restores oxaliplatin sensitivity in Oxa-resistant CRC xenografts. **A** Nude mice were subcutaneously xenografted with SW480 and 480R CRC cells (6 × 10^2^ cells) and treated intraperitoneally with oxaliplatin (5 mg/kg/day per mouse) or DMSO (0.3%) every 5 days. **B** Relative tumor volumes are presented as mean ± SD, *n* = 8. **C** Kaplan–Meier survival curve of nude mice is shown. **D** PiHL levels in SW480 and 480R xenografts were analyzed by qRT-PCR. **E** IHC staining of KLF4, cleaved caspase-3, HMGA2, and p-Akt in consecutive tumor sections of mice subcutaneously xenografted with SW480 and 480R CRC cells. Scale bar = 50 μm. **F** Nude mice were subcutaneously xenografted with 480R CRC cells (6 × 10^2^ cells) transfected with PiHL_LNA or LNA_scramble and treated intraperitoneally with oxaliplatin (5 mg/kg/day per mouse) or DMSO (0.3%) every 5 days. **G** Relative tumor volumes are presented as mean ± SD, *n* = 8. **H** Kaplan–Meier survival curve of nude mice is shown. **I** IHC staining of cleaved caspase-3, HMGA2, and p-Akt in consecutive tumor sections of mice subcutaneously xenografted with 480R CRC cells. Scale bar = 50 μm. *P < 0.05, ***P < 0.001.

To study the role of PiHL in regulating chemotherapeutic resistance of CRC in vivo, we inoculated parental and resistant CRC cells in mice to establish subcutaneous xenografts and examined their drug response to oxaliplatin treatment. After subcutaneous tumor formation, mice received intraperitoneal injections of oxaliplatin (i.p., 5 mg/kg daily) or DMSO every 3 days for 4 weeks (*n* = 8 each group) (Fig. 6A). At the same time, tumors on mice were measured every 5 days. Drug-resistant models exhibited marked limited response toward oxaliplatin treatment and poorer survival (Fig. 6B, C). Consistent with the results mentioned above, Oxa-resistant mice showed decreased levels of KLF4 and cleaved caspase 3, while increased levels of PiHL, HMGA2, and p-AKT by histological analyses (Fig. 6D, E).

To further evaluate the therapeutic potential of PiHL on oxaliplatin-resistant CRC in vivo, we systemically administered an optimized LNA targeting PiHL in a 480R xenograft model (Figs. 6F and S6A). The mice receiving combined treatment of PiHL LNA and oxaliplatin demonstrated a much smaller tumor volume than the other mice (Fig. 6G) and had a significantly prolonged lifespan (Fig. 6H). In addition, immunohistochemistry results demonstrated that, after PiHL silencing, levels of cell apoptosis markers were induced, while HMGA2 and p-AKT expressions were decreased (Fig. 6I). Altogether, these data indicate that PiHL may be a potential therapeutic target for CRC patients to overcome oxaliplatin resistance.

## Discussion

Drug resistance has, to a great extent, limited therapeutic options for CRC patients. Unfortunately, effective treatment strategies to reverse chemoresistance are yet to be established. In recent years, efforts were made to unveil the mystery of lncRNA modulation in CRC biology as well as in oxaliplatin resistance, emphasizing lncRNAs’ potential value as therapeutic intervention targets in cancer therapy. For example, lncRNA linc00152 has been reported to promote oxaliplatin resistance in CRC^25^. LncRNA CCAL was also proven to act as a crucial regulator of chemoresistance in the CRC tumor microenvironment by our previous study^12^. However, the lncRNA-related molecular basis of chemoresistance remains to be elucidated.

Using a series of computational and experimental analyses, our previous research elaborated the critical role of lncRNA PiHL in promoting CRC progression, partially via the p53 signaling pathway^9^. We noticed that PiHL activation could repress cell apoptosis induced by 5-FU in both p53 wild-type and knockout CRC cells, prompting us to further explore PiHL’s potential role in drug resistance and its mechanisms other than p53 regulation.

In this study, we identified a PiHL-induced axis involving the EZH2-mediated activation of PI3K/Akt pathway that contributes to the drug resistance of CRC. We report for the first time that PiHL promotes oxaliplatin resistance in CRC cells using in vitro and in vivo models. We show that lncRNA PiHL is induced in Oxa-resistant CRC cells. We also collected CRC tumor tissues from patients who received oxaliplatin-based therapy after resection and found PiHL high expression to be correlated with poor chemotherapy response. However, these newly collected samples (patients who received tumor resection within 2 years) lack survival data to analyze the relationship between PiHL levels and prognosis of CRC patients, which should be done in the following research.

PRC2 catalyzes methylation of histone H3 lysine 27 (H3K27)^26^. EZH2 is the catalytic subunit of PRC2 and represents a key nuclear target for lncRNAs^27,28^. By interacting with PRC2-EZH2, several lncRNAs including HOTAIR have been reported to bind and change the genomic location of EZH2, therefore epigenetically modulate the expression of target genes involved in various cellular process^23,29^. In this study, we found that lncRNA PiHL was also one of the EZH2-interacting partners. Although we reported that a G-quadruple motif in PiHL is important for binding to EZH2, it remains unclear which part of EZH2 interacts with PiHL. A previous study showed that the N-terminal region of EZH2 is important for RNA binding through a G-rich motif^30^. Given this evidence, the G-quadruple motif embedded in PiHL might also interact with the basic N-terminal helix of EZH2, although such a direct interaction needs to be further verified.

HMGA2 was previously reported as a polycomb group target gene^21,22^. In this study, we further uncovered that via interacting with EZH2, PiHL suppresses the binding of EZH2 to and inhibits H3K27me3 of HMGA2 promoter, resulting in HMGA2 upregulation. Consistently, HMGA2 mRNA levels are positively correlated with PiHL expression in CRC tissues from our cohort. HMGA2 upregulation is known to attenuate cell death under genotoxic drug treatment^19^. In our study, we found that HMGA2 was also able to promote cell survival and oxaliplatin resistance via activating PI3k/Akt pathway. This may help to explain why in p53 knockout CRC cells, overexpression of PiHL, a p53 protein negative regulator, could also suppress apoptosis induced by cytotoxic drug^9^. What has caught our attention in this study is that, in p53 mutant SW480 and HT-29 cells, PiHL dysregulation merely affects cell proliferation under normal conditions, when compared with p53 wild-type HCT116 cells. One possible explanation is that, under normal conditions, p53 signaling dominant in PiHL regulation in p53 wildtype CRC cells, while in p53 mutant cells, HMGA2 plays a superior role in PiHL signaling.

LncRNA PiHL was found to be upregulated in drug-resistant CRC cells, however, neither copy number variation (CNV) nor DNA methylation of *PiHL* gene changes between parental and resistant CRC cells. Transcription factor KLF4 was previously reported to be found primarily in post-mitotic and terminally differentiated epithelial cells of organs such as the gastrointestinal tract^31^. This tumor suppressor is progressively lost in the formation and progression of CRC^32^, but the expression patterns of KLF4 in drug-resistant CRC and its function remain unclear. The current study show, for the first time, that KLF4 has significantly downregulated in oxaliplatin-resistant cells. Here we also provide evidence that induced PiHL expression in resistant cells can be contributed by KLF4 downregulation, PiHL is a downstream target of KLF4 and KLF4 suppresses PiHL expression transcriptionally. These results indicate KLF4’s potential suppression role in drug resistance. Accumulating evidence suggests that low KLF4 mRNA levels could be attributed to hemizygous deletion, point mutations, or hypermethylation of 5′-untranslated regions of the KLF4 gene. Besides, indirect factors such as β-catenin activation, APC mutations, and microRNA regulation could also lead to KLF4 loss^33–36^. However, the underlying molecular mechanism for KLF4 low expression in chemoresistant CRC needs to be further explored.

**Fig. 7:**
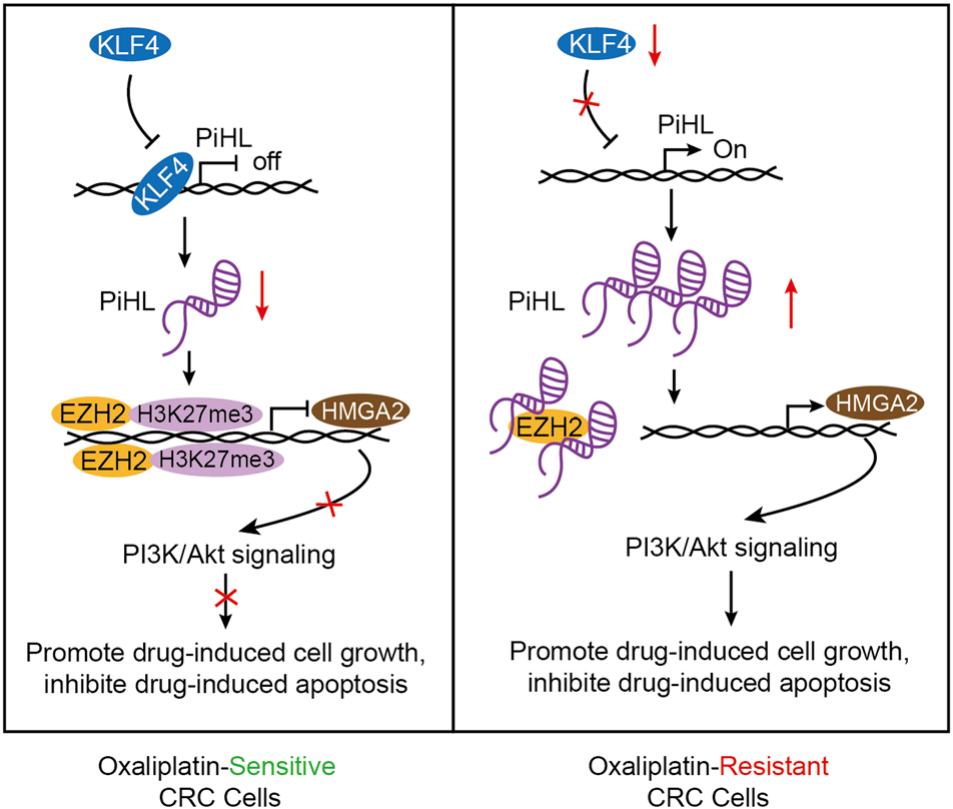
A proposed schematic model of PiHL regulating oxaliplatin resistance in CRC. KLF4 Krüppel-like factor 4, PiHL P53 inHibiting LncRNA, EZH2 Enhancer of zeste 2, H3K27me3 methylation of histone H3 lysine 27, HMGA2 highmobility group A2, CRC colorectal cancer.

In summary, PiHL is induced by KLF4 downregulation and confers chemoresistance to tumor cells. PiHL physically interacts with EZH2 to modulate H3K27me3 of HMGA2, resulting in PI3K/Akt activation. LncRNA PIHL may serve as a therapeutic target to overcome oxaliplatin resistance, enhancing the clinical benefits of oxaliplatin chemotherapy in CRC patients (Fig. 7).

## Supplementary information

Supplementary information
