# Supplementary material for: A KLF4/PiHL/EZH2/HMGA2 regulatory axis and its function in promoting oxaliplatin-resistance of colorectal cancer
Source: Cell Death Dis. 2021 May 13;12(5):485. doi: 10.1038/s41419-021-03753-1 (PMC8119946; doi:10.1038/s41419-021-03753-1)
Supplement: Supplementary file 1 — Supplementary information [file 41419_2021_3753_MOESM1_ESM.pdf]

Supplementary Information:

This PDF includes:

Supplementary Figure Legends

Supplementary Figure S1-6.

Supplementary Table 1 and 2

Supplementary Figure Legends:

Supplementary Figure 1 The establishment of Oxa-resistant CRC cells. (A) Schematic process to acquire Oxa-resistant CRC cells. (B) CCK-8 assay of Oxa-resistant and parental CRC cells upon oxaliplatin treatment at the indicated concentrations for 72 hours. (C) Colony formation assay of Oxa-resistant and parental CRC cells upon oxaliplatin treatment (5 μM) in a 6-well dish (500 cells per well) for 14 days. The average number of colonies are shown. (D) Flow cytometric analysis of apoptosis in Oxa-resistant and parental CRC cells upon oxaliplatin treatment (5 μM) for 72 hours. The average number of apoptotic cells are shown. Data are presented as the mean ± S.D, *P < 0.05, **P < 0.01, ***P < 0.001.

Supplementary Figure 2 Copy number and DNA methylation alterations of PiHL. (A) qRT-PCR analysis of PiHL genomic copy numbers in Oxa-resistant and parental CRC cells. (B) qRT-PCR analysis of PiHL in parental CRC cells treated with azacitidine (5 μM or 10 μM) or DMSO. (C) KLF4 knockdown efficiencies were examined by qRT-PCR and immunoblots. (D) Levels of PiHL in KLF4 silencing HT-29 cells were analyzed by qRT-PCR. Data are presented as the mean ± S.D. P value was determined by Student's t test. Significant results were presented as n.s. (non-significant), **P < 0.01, ***P < 0.001.

Supplementary Figure 3 PiHL overexpression induces oxaliplatin resistance in CRC cells *in vitro*. (A) IC50 values of oxaliplatin in CRC cells with overexpression of ABCA4, ABCB6, ABCA9, ABCA10 or control were determined using the CCK-8 assay. **(**B, C) PiHL knockdown (B) and overexpression (C) efficiencies in oxaliplatin resistant and parental CRC cells were examined by qRT-PCR. (D, E) Expression of cleaved Caspase-3 (C-Caspase-3) and cleaved PARP (C-PARP) in PiHL silencing HT-29 cells (D) and PiHL overexpression CRC cells (E) after 5 μM oxaliplatin treatment for 72 hours were analyzed by western blotting. (F) Effect of PiHL knockdown on HT-29 cells with oxaliplatin treatment at the indicated concentrations for 72 hours was analyzed by CCK-8 assay. (G) Flow cytometric analysis (left), colony formation assay (middle) and EdU assay (right) were performed in PiHL knockdown or control HT-29 cells with oxaliplatin treatment (5 μM) for 72 hours. (H) Effect of PiHL overexpression on CRC cells with oxaliplatin treatment at the indicated concentrations for 72 hours was analyzed by CCK-8 assay. (I) Flow cytometric analysis (left), colony formation assay (middle) and EdU assay (right) were performed in PiHL overexpressed or control CRC cells with oxaliplatin treatment (5 μM) for 72 hours. (J) Western blot analysis of indicated proteins in HT-29 cells with PiHL knockdown or controls. P value was determined by Student's t-test or one-way ANOVA. Significant results were presented as n.s. non-significant or *P < 0.05, **P < 0.01, ***P < 0.001.

Supplementary Figure 4 PiHL targeting HMGA2 is required for PiHL-mediated CRC drug resistance. (A) HMGA2 levels regulated by PiHL in HT-29 cells was confirmed by qRT-PCR (left) and western blotting (right). (B) HMGA2 knockdown efficiencies were examined by qRT-PCR (left) and western blotting (right). (C) CCK-8 (left), flow cytometry (middle) and colony formation assay (right) were used to determine cell survival, apoptosis and growth of HT-29 cells with HMGA2 knockdown treated with 5 μM oxaliplatin for 72 hours. (D) CCK-8 (left), flow cytometry (middle) and colony formation assay (right) were used to determine cell survival, apoptosis and growth of HMGA2 overexpressed CRC cells treated with 5 μM oxaliplatin for 72 hours. (E) CCK-8 (left), flow cytometry (middle) and colony formation assay (right) were used to determine cell survival, apoptosis and growth of HT-29 cells under shNC+Vector, shPiHL+Vector or shPiHL+HMGA2 conditions treated with 5 μM oxaliplatin for 72 hours. (F) Levels of HMGA2 and PI3K/Akt signaling were tested by western blotting in HT-29 cells with indicated treatment. Data are presented as the mean ± S.D. *P < 0.05, **P < 0.01, ***P < 0.001.

Supplementary Figure 5 PiHL interacts with EZH2 via a G-quadruple motif. (A) Immunoblots of HMGA2 proteins retrieved by in-vitro-transcribed biotinylated PiHL from cell nuclear extracts. Antisense PiHL and beads were used as negative controls. (B) Two putative G-quadruple regions in PiHL transcripts. (C) Relative PiHL expression determined using the two primer sets referred to (B) in EZH2-IP. (D) ChIP assay using EZH2 and H3K27me3 specific antibodies was undertaken in PiHL stably knockdown Oxa-resistant CRC cells to detect the effects of PiHL on EZH2 location and H3K27me3 level at *GAPDH* promoter. (E) ChIP assay using EZH2 and H3K27me3 specific antibodies was undertaken in PiHL stably knockdown HT-29 cells to detect the effects of PiHL on EZH2 location and H3K27me3 level at *HMGA2* (left) and *GAPDH* (right) promoter. (F) ChIP assay using EZH2 (left) and H3K27me3 (right) specific antibodies was undertaken in PiHL stably overexpressed CRC cells to detect the effects of PiHL on EZH2 location and H3K27me3 level at *GAPDH* promoter. (G) HMGA2 RNA (left) and protein (right) levels were measured in HCT116 cells transfected with pCDH (Vector), pCDH-PiHL (PiHL), or PiHL-Mut by qRT-PCR and immunoblot, respectively. Data represent the mean ± SD from 3 independent experiments. *P < 0.05, **P < 0.01, ***P < 0.001.

Supplementary Figure 6 (A) PiHL levels in 480R xenografts treated with PiHL_LNA or LNA_scramble were analyzed by qRT-PCR. Data are presented as the mean ± S.D. P value was determined by Student's t test, **P < 0.01.

Figure S1

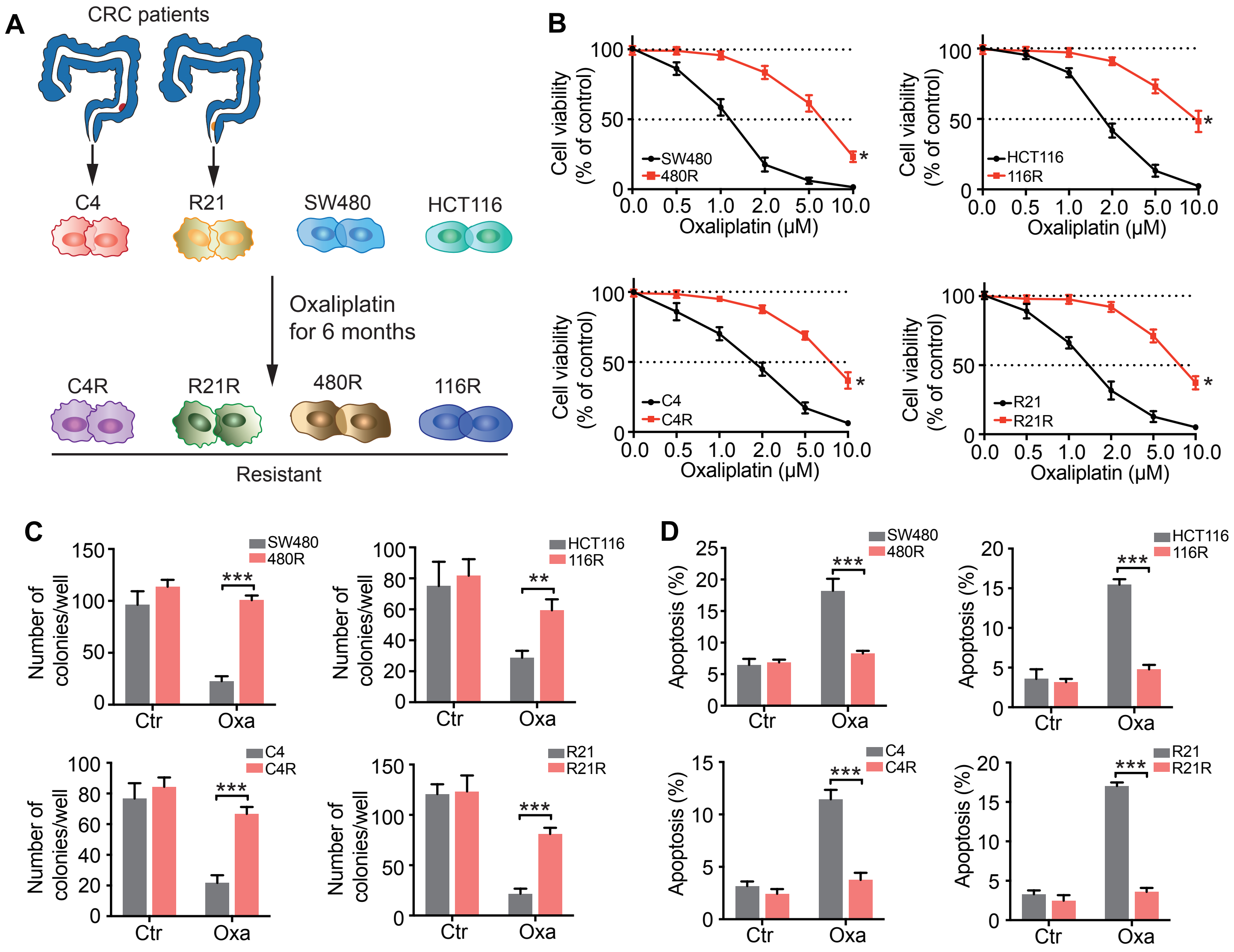
A
CRC patients
C4
R21
SW480
HCT116
Oxaliplatin
for 6 months
C4R
R21R
480R
116R
Resistant
B
Cell viability
(% of control)
Oxaliplatin (μM)
0.0 0.5 1.0 2.0 5.0 10.0
SW480
480R
HCT116
116R
C4
C4R
R21
R21R
*
C
Number of
colonies/well
Ctr
Oxa
***
**
D
Apoptosis (%)
Ctr
Oxa
***

Figure S2

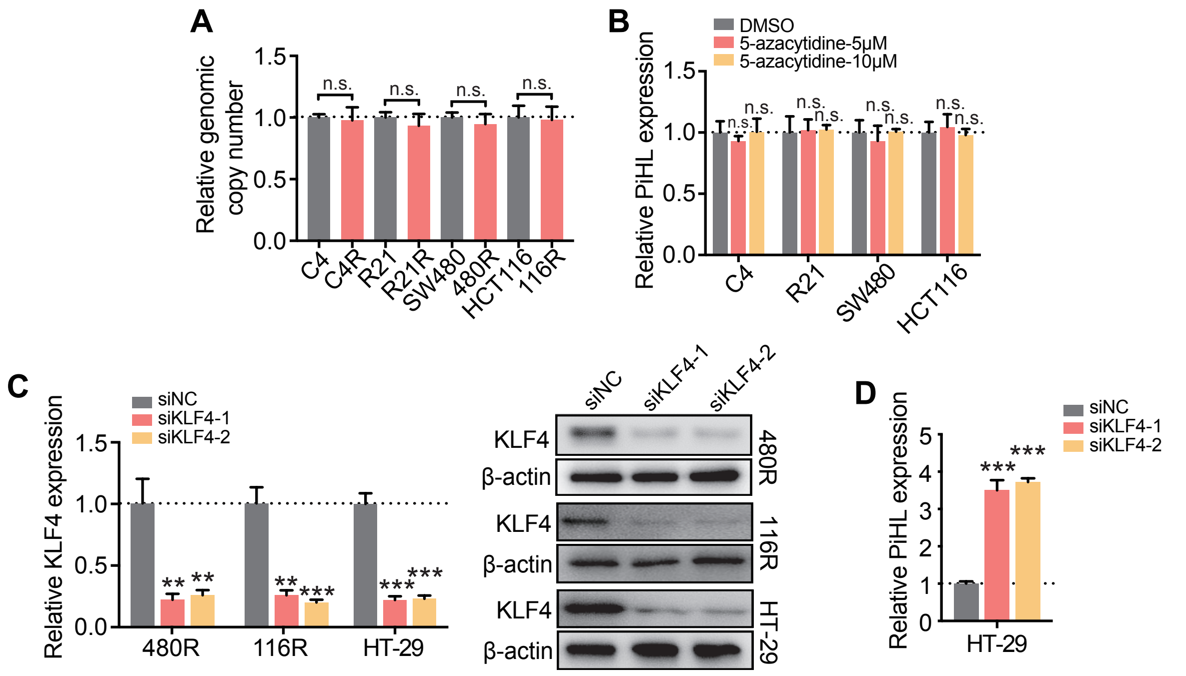
A
Relative genomic copy number
1.5
1.0
0.5
0.0
n.s.
n.s.
n.s.
n.s.
C4
C4R
R21
R21R
SW480
480R
HCT116
116R
B
DMSO
5-azacytidine-5μM
5-azacytidine-10μM
Relative PiHL expression
1.5
1.0
0.5
0.0
n.s.
C4
R21
SW480
HCT116
C
siNC
siKLF4-1
siKLF4-2
Relative KLF4 expression
1.5
1.0
0.5
0.0
**
***
480R
116R
HT-29
siNC
siKLF4-1
siKLF4-2
KLF4
β-actin
480R
116R
HT-29
D
siNC
siKLF4-1
siKLF4-2
Relative PiHL expression
5
4
3
2
1
0
***
HT-29

Figure S3

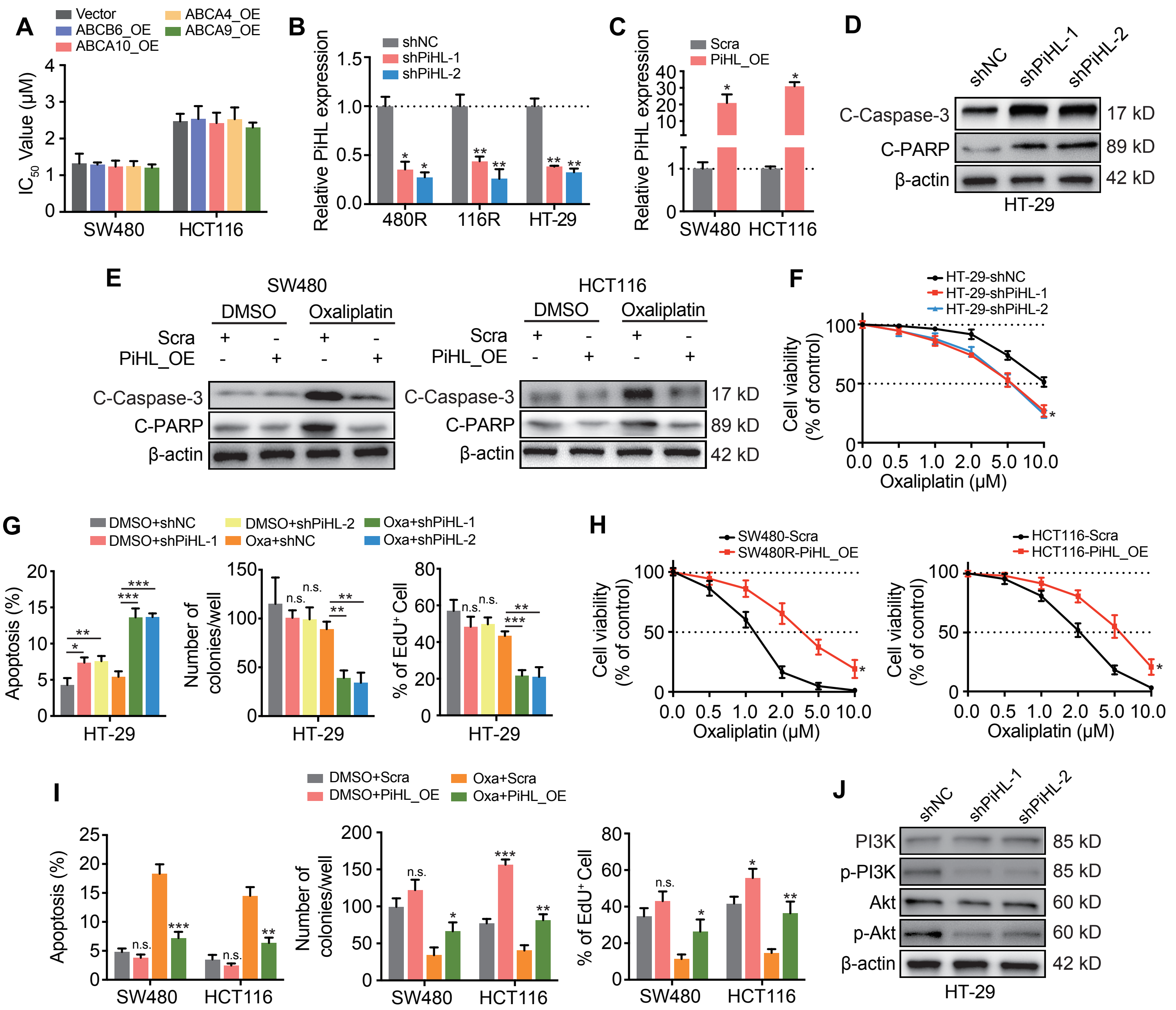

A
Vector
ABCB6_OE
ABCA10_OE
ABCA4_OE
ABCA9_OE
IC50 Value (μM)
SW480
HCT116
B
shNC
shPiHL-1
shPiHL-2
Relative PiHL expression
480R
116R
HT-29
C
Scra
PiHL_OE
Relative PiHL expression
SW480
HCT116
D
shNC
shPiHL-1
shPiHL-2
C-Caspase-3
C-PARP
β-actin
17 kD
89 kD
42 kD
HT-29
E
SW480
HCT116
DMSO
Oxaliplatin
Scra
PiHL_OE
C-Caspase-3
C-PARP
β-actin
17 kD
89 kD
42 kD
F
HT-29-shNC
HT-29-shPiHL-1
HT-29-shPiHL-2
Cell viability (% of control)
Oxaliplatin (μM)
G
DMSO+shNC
DMSO+shPiHL-1
DMSO+shPiHL-2
Oxa+shNC
Oxa+shPiHL-1
Oxa+shPiHL-2
Apoptosis (%)
Number of colonies/well
% of EdU+ Cell
HT-29
H
SW480-Scra
SW480R-PiHL_OE
HCT116-Scra
HCT116-PiHL_OE
Cell viability (% of control)
Oxaliplatin (μM)
I
DMSO+Scra
DMSO+PiHL_OE
Oxa+Scra
Oxa+PiHL_OE
Apoptosis (%)
Number of colonies/well
% of EdU+ Cell
SW480
HCT116
J
shNC
shPiHL-1
shPiHL-2
PI3K
p-PI3K
Akt
p-Akt
β-actin
85 kD
85 kD
60 kD
60 kD
42 kD
HT-29

Figure S4

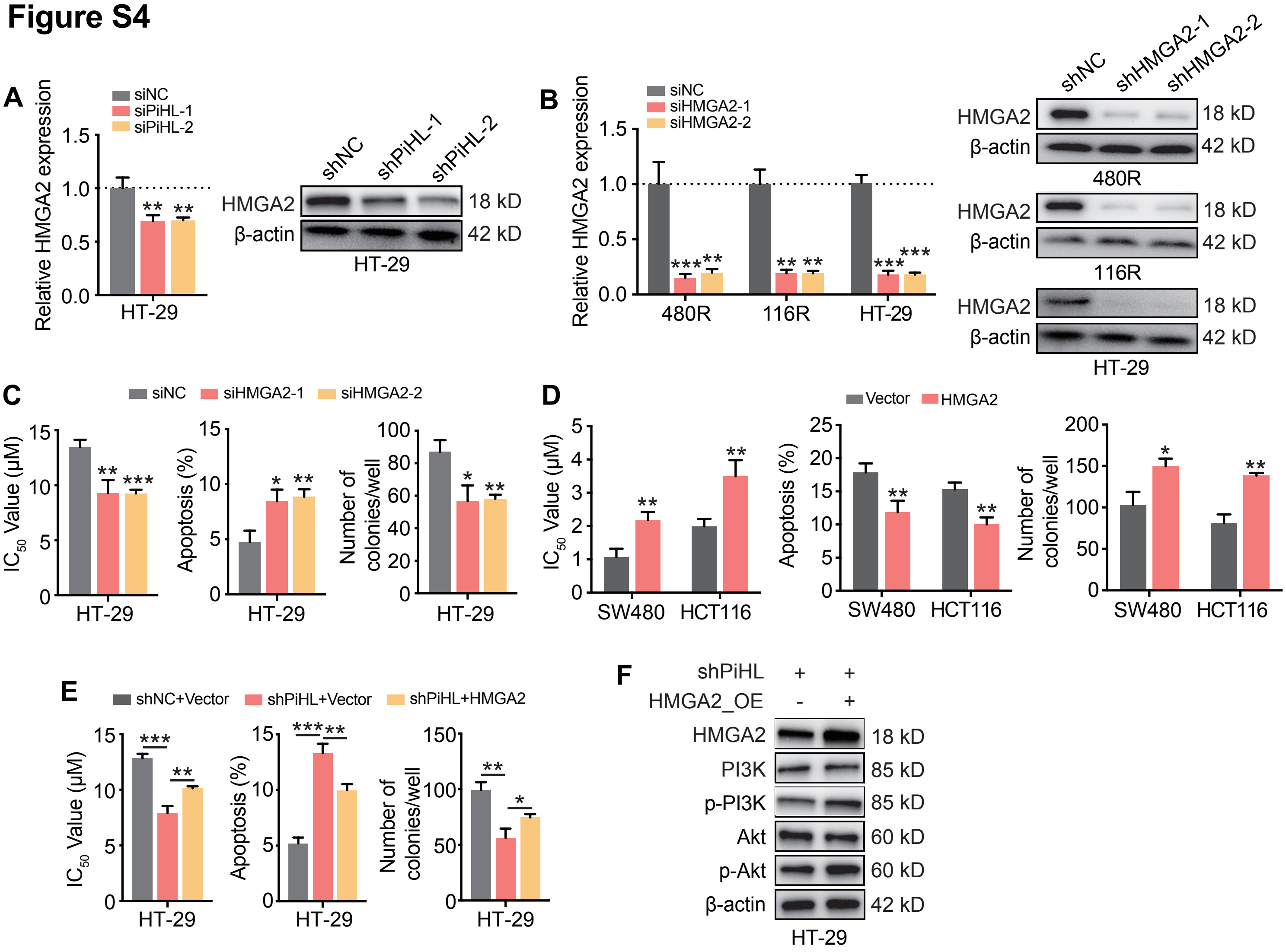
A
siNC
siPiHL-1
siPiHL-2
Relative HMGA2 expression
HT-29
shNC
shPiHL-1
shPiHL-2
HMGA2
β-actin
18 kD
42 kD
HT-29
B
siNC
siHMGA2-1
siHMGA2-2
Relative HMGA2 expression
480R
116R
HT-29
shNC
shHMGA2-1
shHMGA2-2
HMGA2
β-actin
18 kD
42 kD
480R
116R
HT-29
C
siNC
siHMGA2-1
siHMGA2-2
IC50 Value (μM)
Apoptosis (%)
Number of colonies/well
HT-29
D
Vector
HMGA2
IC50 Value (μM)
Apoptosis (%)
Number of colonies/well
SW480
HCT116
E
shNC+Vector
shPiHL+Vector
shPiHL+HMGA2
IC50 Value (μM)
Apoptosis (%)
Number of colonies/well
HT-29
F
shPiHL
HMGA2_OE
HMGA2
PI3K
p-PI3K
Akt
p-Akt
β-actin
18 kD
85 kD
85 kD
60 kD
60 kD
42 kD
HT-29

Figure S5

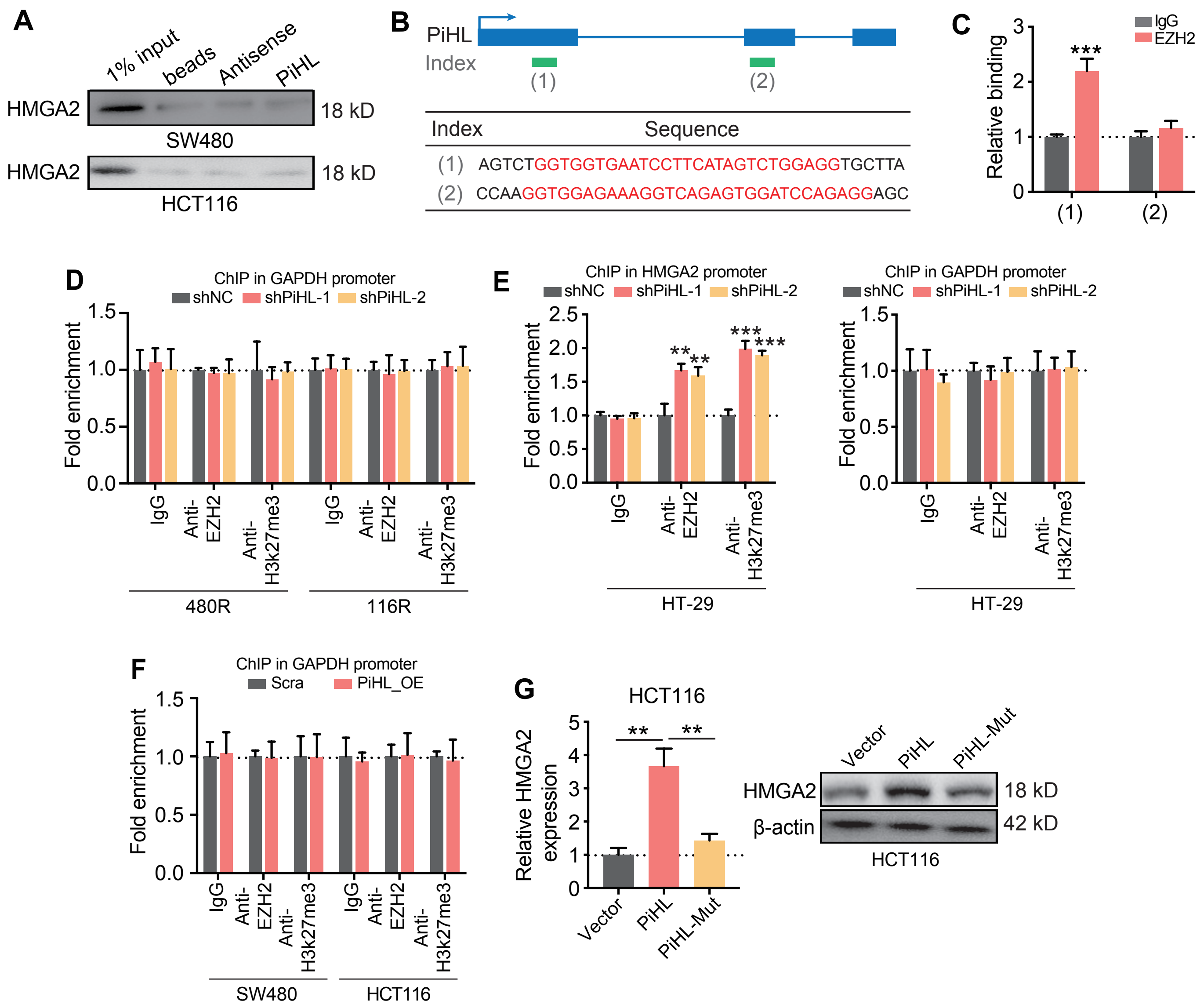

A
1% input
beads
Antisense
PiHL
HMGA2
18 kD
SW480
HMGA2
18 kD
HCT116
B
PiHL
Index
(1)
(2)
Index
Sequence
(1) AGTCTGGTGGTGAATCCTTCATAGTCTGGAGGTGCTTA
(2) CCAAGGTGGAGAAAGGTCAGAGTGGATCCAGAGGAGC
C
IgG
EZH2
Relative binding
***
D
ChIP in GAPDH promoter
shNC
shPiHL-1
shPiHL-2
Fold enrichment
IgG
Anti-EZH2
Anti-H3k27me3
480R
116R
E
ChIP in HMGA2 promoter
ChIP in GAPDH promoter
HT-29
HT-29
F
ChIP in GAPDH promoter
Scra
PiHL_OE
SW480
HCT116
G
HCT116
Relative HMGA2 expression
Vector
PiHL
PiHL-Mut
HMGA2
β-actin
18 kD
42 kD
HCT116

Figure S6

A

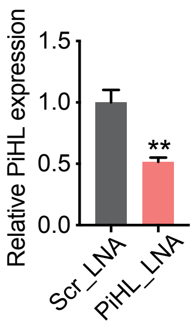

Supplementary Tables:

Supplementary Table S1. Primers used in this study.

**Primers used for quantitative RT-PCR**		
Name	Forward	Reverse
lncRNA-PiHL	GAGCCAAGAGAAGACGTCCAG	AAAGGCCAACAGGAACCACAT
GAPDH	TCACCACCATGGAGAAGGC	GCTAAGCAGTTGGTGGTGCA
β-actin	AGTTGCGTTACACCCTTTCTTG	GCTGTCACCTTCACCGTTCC
HMGA2	GGTGCAAGACTCAGGAGCTA	AGTCGGAAAGCAAAGGAGGA
HOTAIR	GGAAGCGAAGGGGTTGTGTA	GGCTAGGGCTGGTTTCACTT
KLF4	ATGCTCACCCCACCTTCTTC	TTCTCACCTGTGTGGGTTCG
**Primers used for ChIP-qPCR**	Forward	Reverse
HMGA2 promoter	TTCCCACTCACAGTGAACCG	CTTTACCTGCGCCTCTACCG
GAPDH promoter	CACAGTCCAGTCCTGGGAAC	TAGTAGCCGGGCCCTACTTT
PiHL distant region	GACACCCACCATCATCCAGG	GGGCACATGTCCAAACCAAC
PiHL promoter	GGAAGGGGAGGGGTGA	GCCCTTTGGCTCAAGGAACACA
PiHL exon 2	ATGCAGTGTCCAAGGTGGAG	AAAGGCCAACAGGAACCACA
**Primers used for PiHL-Mut construction**	Forward	Reverse
PiHL-Mut	TGCTTATTTAGCAAATTCAACCTTAAAC	CGAGGAGCAGTCTCCTGA

Supplementary Table S2. Sequences of RNAi, antisense, and probes used in this study

Name	Target sequence
siRNA-lncRNA-PiHL-1	CGCCAAAGCUUCAGGAGACU
siRNA-lncRNA-PiHL-2	GAGAAGACGUCCAGCATGGU
siRNA-NC	UCCUAAGGUUAAGUCGCCCUC
siRNA-KLF4-1	GAAUUGGACCCGGUGUACAUU
siRNA-KLF4-2	GGUGAGAAACCUUACCACTGU
siRNA-HMGA2-1	GAUAAGGACUAGAUACUAC
siRNA-HMGA2-2	AUGGAAGCAAUUGCUCAUG
PiHL FISH probe	UCUGGUGGUGAAUCCUUCAUAGUCUGGA
Scra_LNA	UCCUAAGGUUAAGUCGCCCUC
PiHL_LNA	CGCCAAAGCUUCAGGAGACU
